# Measurement of the Corpus Callosum Using Magnetic Resonance Imaging in the North of Iran

**DOI:** 10.5812/iranjradiol.4495

**Published:** 2011-12-25

**Authors:** Mohammad Reza Mohammadi, Pouya Zhand, Behnoush Mortazavi Moghadam, Mohammad Jafar Golalipour

**Affiliations:** 1 Department of Neurosurgery, 5 Azar Hospital, Golestan University of Medical Sciences, Gorgan, Iran; 2 General physician and Researcher, 5 Azar Hospital, Golestan University of Medical Sciences, Gorgan, Iran; 3 Department of Radiology, 5 Azar Hospital, Golestan University of Medical Sciences, Gorgan, Iran; 4 Department of Anatomical Sciences, Dezyani Hospital, Golestan University of Medical Sciences, Gorgan, Iran

**Keywords:** Brain, Corpus Callosum, Sex, Magnetic Resonance Imaging, Iran

## Abstract

**Background:**

Morphometric measurements of the corpus callosum (CC) are important to have normative values according to sex, age and race/ethnicity.

**Objectives:**

This study was done to measure the size of CC and to identify its gender- and age-related differences in the North of Iran.

**Patients and Methods:**

The size of CC on midsagittal section was measured in 100 (45 males, 55 females) normal subjects using magnetic resonance imaging (MRI) admitted to the Kowsar MRI center in Gorgan–Northern Iran.

Longitudinal and vertical dimensions of the CC, longitudinal and vertical lengths of the brain and the length of genu and splenium were measured. Data were analyzed by student’s unpaired t test, ANOVA and regression analysis.

**Results:**

The anteroposterior length and vertical dimension of the CC, the length of genu and splenium were larger in males than in females, but these differences were not significant. The anteroposterior and vertical lengths of the brain were significantly larger in males than in females (P < 0.05). The length of CC increased with age and regression equations for predicting age were derived from the length of the CC. There was also a positive significant correlation between the anteroposterior length of the CC and the length of the brain and vertical dimension of the CC.

**Conclusions:**

This study showed that various CC parameters vary with the values documented in the Caucasian, Indian and Japanese population.

## 1. Background

The corpus callosum (CC) with more than 300 million fibers is the most important anatomical and functional inter-hemispheric commissure in the human brain. The activities of the left and right cerebral hemispheres correlate via CC. Callosal fibers have a special topographic organization [1]. It means that the prefrontal cortex of the right and left human cerebral hemispheres correlate via genu and rostrum. Besides, premotor and supplementary motor cortical areas, primary motor and primary sensory cortex of the two hemispheres link via the body of the CC; whereas, parietal, temporal and occipital fiber bundles of the two hemispheres cross the CC through the splenium.

CC has a special influence on the affective behavior, non-literal language and bilateral functional connection in both motor and sensory cortices [2][3]. Several studies have reported that the size of CC changes in bipolar disorders [4], Alzheimer disease [5], leukoaraiosis [6] and Williams syndrome [7]. Indeed, some researchers have reported that the morphology of CC may change in diseases such as dyslexia [8], Tourette’s syndrome [9], Down’s syndrome [10], depression [11], schizophrenia [12] and HIV/AIDS [13].

Regarding differences in the size of organs in humans including CC according to race/ethnicity in various parts of the world, CC dimensions, morphology and sex-related differences have been of interest to researchers [1].

By using magnetic resonance imaging (MRI), the dimensions of CC including size, diameters, age morphology and also gender-related differences have been determined in several studies [1][14][15][16][17][18][19][20][21][22][23][24][25][26].

Most of the studies on the size and shape of CC were carried out in Western countries on the Caucasian population [1][14][15][16][17][18] and a few studies were performed in the East Asian [21][23] and Indian population [19][20][22].

Minimal variability in the dimensions and relative dimensions of the CC in Greek people was reported by Mourgila et al. [1]. They also found that the longitudinal dimension of the genu (EZ/3) and total CC (EZ) are larger in males.

In another study carried out by Gupta et al. on Indians, no sexual dimorphism was observed in most of the CC parameters. They also reported that shrinkage of the anterior half of the CC due to aging could be due to the atrophic alteration of the brain [19]. One other of their findings was that the length and width of CC in Indians were less than those of the Caucasian population [22].

Takada in 2003 did not observe any difference in the regional size of CC between genders in Japanese subjects [21]. On the other hand, Bermudez and Zatorre [15] found a well established difference in size, shape and position of the CC between genders.

Furthermore, Luders et al. [16] did not find significant sex difference in scaled data. Although few studies have been performed in different parts of the world on the size and shape of CC reporting different findings about it, there is no documented data regarding anatomical characteristics of CC in the Iranian population.

## 2. Objectives

This study was performed to determine the longitudinal and vertical dimensions of the brain and the dimensions of various parts of CC by MRI.

## 3. Patients and Methods

This descriptive study was carried out on 100 subjects (45 male) admitted to Kowsar MRI center in Gorgan–Northern Iran from 24 July, 2006 to 16 December, 2006. Informed consent was obtained along with a clearance from the institutional ethics committee.

The subjects were referred to Kowsar MRI center for headache survey. Inclusion criteria were no neurological signs, no intracranial lesions, mass or head injury on MRI and no history of neurological disease. The subjects were divided into three age groups including less than 30 years old, 30-44 and 45 years old and more.

**Figure 1:**
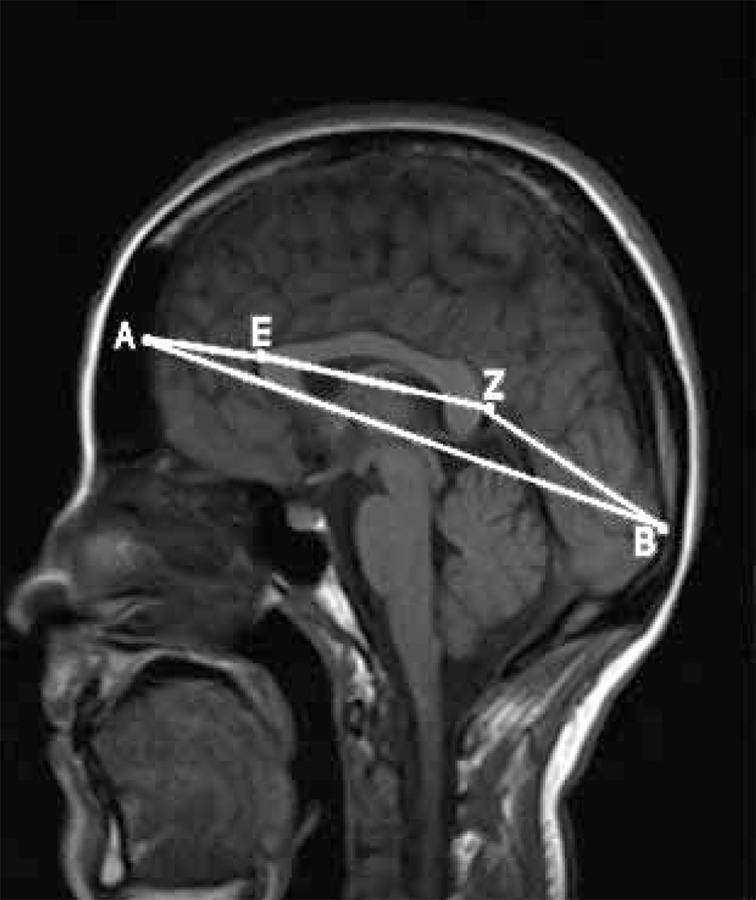
Brain MRI demonstrating the measured dimensions. A, Frontal pole of the brain; B, Occipital pole of the brain; E, Geno of the corpus callosum; Z, Splenium of the corpus callosum

Brain and CC dimensions were measured on MRI unit (Siemens, symphony, 1.5 Tesla). MR images were acquired in the axial and vertical planes by using FLAIR, T1 and T2 weighted sequences. For each case, using a mid-sagittal view of the cerebral hemispheres, the following global dimensions [1][22] were measured (Figure 1):

AB: Longitudinal dimension of the brain from the frontal to the occipital pole

CD: From the superior to the inferior surface of the brain, including the cerebellum

AE: From the frontal pole of the brain to the genu of CC

BZ: From the occipital pole of the brain to the splenium of CC

EZ: Length of CC from anterior most point of CC to its posterior most point. According to the maximum straight length by Witelson’s method [1] EZ is subdivided into three regions:

1) The genu (EZ/3) was defined as the anterior third.

2) The mid-body of the CC was allocated the middle third.

3) The posterior third was subdivided into A) the posterior fifth (splenium-EZ/5) and B) the isthmus, a region between the mid body and the splenium.

FG considered as the width of CC in the vertical view of the brain hemispheres. The width of CC was measured at the midpoint, which was taken at the center of the CC length.

The various dimensions of the CC (AB, CD, AE, BZ, EZ, EZ/3, EZ/5 and FG) were correlated with each other. Then, these dimensions were correlated with the dimensions of the brain. These findings were then examined for possible sex- and age-related differences.

Statistical analysis was conducted using SPSS version 11.5 and ANOVA, TUKEY and t student tests. For all the comparisons P lower than 0.05 was considered as significant.

The 95% confidence interval (CI) for variables was calculated. For determining correlation of EZ as a dependent variable and AB, AE and FG as independent variables, we used multi variant linear regression.

Pearson correlation test was used to determine linear correlation between quantitative variables.

## 4. Results

The mean value for the longitudinal dimension of the brain (AB) was 16.12 ± 0.081 cm (95% CI; 15.96-16.28), while the mean value for the longitudinal dimension of the CC (EZ) was 7.06 ± 0.052 cm (95% CI; 6.96-7.17), a ratio greater than 2:1.

The mean value for the longitudinal dimension of the genu (EZ/3) and the splenium (EZ/5) was 2.35 ± 0.017 cm (95% CI; 2.316-2.385) and 1.41 ± 0.010 cm (95% CI; 1.388-1.429), respectively.

The distance between the genu and the frontal pole (AE) had a mean value of 3.68 ± 0.034 cm (95% CI; 3.616-3.751), while the distance from the splenium to the occipital pole (BZ) was 5.580 ± 0.052 cm (95% CI; 5.476-5.485), an approximate ratio of 1:1.5.

The mean value for the distance between the upper and lower surfaces of the brain (CD-vertical diameter) was 10.63 ± 0.51 cm.

A positive linear correlation was evident between AB and CD (r = 0.45), AB and AE (r = 0.45), and between AB and EZ (r = 0.56). A stronger positive correlation (r = 0.71) was noted between AB and BZ (the distance between the splenium and the occipital pole). CD (vertical brain diameter) exhibited a positive linear correlation with AB (r = 0.45), but not with EZ (r = 0.04) (Table 1).

**Table 1:** Spearman’s Rho Correlation Coefficient Between the Corpus Callosum Diameters (n = 100)

**Morphometric Index**	**AE**	**BZ**	**CD**	**FG**	**EZ**^a^
AB^a^, cm	0.449 b	0.713 b	0.448 b	0.122	0.560 b
AE^a^, cm	-	0.314 b	0.428 b	-0.160	-0.252
BZ a, cm	-	-	0.403 b	0.081	0.034
CD a, cm	-	-	-	0.235 c	0.041
FG a, cm	-	-	-	-	0.236 c

^a^ AB, Longitudinal dimension of the brain from the frontal to the occipital pole; AE, From the frontal pole of the brain to the genu of CC; BZ, From the occipital pole of the brain to the splenium of CC; CD, From the superior to the inferior surface of the brain, including the cerebellum; EZ, Length of CC from anterior most point of CC to its posterior most point; FG, Considered as the width of CC in the vertical view of the brain hemispheres.

^b^ P < 0.05

^c^ P < 0.01

Multi variant linear regression showed that there is a significant correlation between EZ (as the dependent variable of the model) and AB and AE (P < 0.0001) (Table 2). The results have also shown that FG had no significant correlation with EZ in the mentioned multivariant linear regression model.

**Table 2:** Multivariant Linear Regression Between EZ and AB, AE and FG

**Model** ****	**Unstandardized Coefficients**		**Standardized Coefficients**	**T value** ****	***P* value**
	**B**	**Standard-Error**	**Beta**		
Constant	1.766	0.667	-	2.647	0.009
AB a, cm	0.536	0.045	0.835	11.780	< 0.0001
AE^a^, cm	-0.943	0.108	-0.622	-8.717	< 0.0001
FG^a^, cm	0.237	0.439	0.035	0.539	0.591

^a^ AB, Longitudinal dimension of the brain from the frontal to the occipital pole; AE, From the frontal pole of the brain to the genu of CC; FG, Considered as the width of CC in the vertical view of the brain hemispheres.

Regarding sex-related differences, the mean of the longitudinal dimensions and measured ratios tended to be smaller in women, and the inter-sex difference in mean value for AB (male; = 16.46 ± 0.11 cm, female; = 15.83 ± 0.10 cm; P < 0.001), AE (male; = 3.77 ± 0.05 cm, female; = 3.60 ± 0.04 cm, P < 0.012), BZ (male; = 5.81 ± 0.08 cm, female; = 5.40 ± 0.06 cm, P < 0.001) and CD (male; = 10.82 ± 0.08 cm, female; = 10.47 ± 0.06 cm, P < 0.01) was statistically significant (Table 3).

**Table 3:** Dimensions of Corpus Callosum in the Iranian Population According to Gender

**Morphometric Index**	**Female, Mean ± SEM **d	**Male, Mean ± SEM **d	**T value**	***P* value**
AB a	15.83 ± 0.10	16.46 ± 0.11	4.21	< 0.0001
EZ a	7.03 ± 0.07	7.10 ± 0.07	0.62	0.535
AE a	3.60 ± 0.04	3.77 ± 0.05	2.57	0.012
BZ a	5.40 ± 0.06	5.81 ± 0.08	4.07	< 0.0001
EZ/3 b	2.34 ± 0.02	2.36 ± 0.02	0.64	0.523
EZ/5 c	1.40 ± 0.01	1.41 ± 0.01	0.61	0.540
CD a	10.47 ± 0.06	10.82 ± 0.08	3.60	0.001
FG a	0.54 ± 0.01	0.56 ± 0.01	1.55	0.123

^a^ These are explained in the (first footnote) of Table 1

^b^ EZ/3, Longitudinal dimension of the genu

^c^ EZ/5, The posterior fifth

^d^ Abbreviation: SEM, standard error of mean

**Figure 2:**
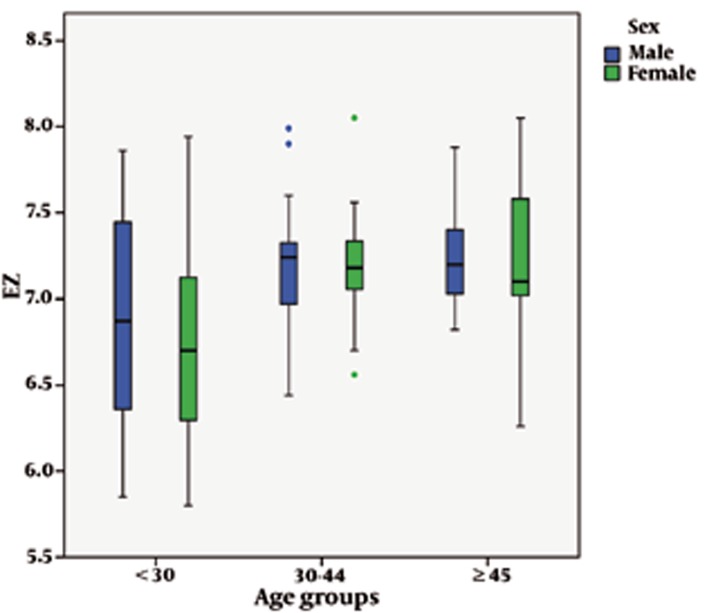
The graph shows dimensions of EZ of the corpus callosum according to gender and age groups.

**Figure 3:**
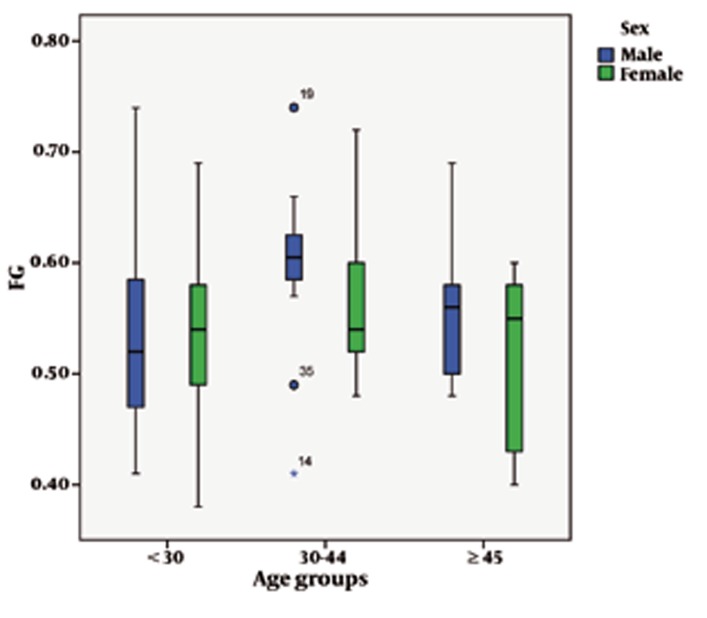
The graph shows dimensions of FG of the corpus callosum according to gender and age groups.

Regarding age, there was a statistically-significant increase in the longitudinal dimensions of the CC. The mean length of the CC was 7.25 ± 0.08 cm in those subjects who were 45 years old and more. It was 7.19 ± 0.06 cm in those who were 30-44 years old while in those younger than 30 years it was 6.82 ± 0.10 cm (P = 0.001) (Table 4). ANOVA and Tukey tests showed that mean of EZ was significantly increased in 30-44 and ≥ 45 age groups compared to < 30 age group and the mean of FG was significantly increased in 30-44 age group compared to < 30 age and ≥ 45 age groups (Figure 2 and 3 and Table 4).

**Table 4:** Dimensions of Corpus Callosum in the Iranian Population According to Age

**Morphometric Index**	** Age, y, Mean ± SEM b**			**F value**	***P* value**
	**< 30**	**30-44**	**≥ 45**		
AB a	16.13 ± 0.14	16.05 ± 0.13	16.18 ± 0.13	0.2	0.795
EZ a	6.82 ± 0.10	7.19 ± 0.06	7.25 ± 0.08	8.1	0.001
AE a	3.88 ± 0.05	3.59 ± 0.05	3.51 ± 0.06	13.7	< 0.0001
BZ a	5.65 ± 0.09	5.51 ± 0.10	5.61 ± 0.09	0.7	0.512
EZ/3 a	2.27 ± 0.03	2.39 ± 2.02	2.41 ± 0.03	8.3	< 0.0001
EZ/5 a	1.36 ± 0.02	1.43 ± 0.01	1.45 ± 0.02	8.1	0.001
CD a	10.82 ± 0.08	10.64 ± 0.07	10.33 ± 0.09	8.3	< 0.0001
FG a	0.53 ± 0.01	0.58 ± 0.01	0.53 ± 0.01	4.2	0.018

^a^ These are explained in the (first footnote) of Table 3

^b^ Abbreviation: SEM, standard error of mean

## 5. Discussion

According to our findings there is no significant sexual dimorphism in human CC dimensions in the North of Iran, although the dimensions of CC in males was higher than females. Besides, there was a positive correlation between CC and brain longitudinal diameters.

In recent years, several studies on MRI scans have been carried out to determine the diameters, morphology and sex-related differences of CC in various parts of the world [1][14][15][16][17][18][19][20][21][22][23][24][25][26].

In a study by Takeda et al. (2003) using MRI on the Japanese showed that the length and height of CC was 69.7 ± 4.15 and 25.9 ± 2.90 mm, respectively in males and 69.4 ± 4.33 and 25.8 ± 2.80 mm, respectively in females. He concluded that there is no difference in callosal measures between the two genders [21].

Bermudez and Zatorre in a study on 137 young normal volunteers reported that male subjects show significantly larger absolute total areas as the anterior third and posterior midbody of CC. However, the total area of the anterior midbody and splenium in females were bigger than males. Moreover, a strong difference in size, shape and position of CC among genders were found [15].

In Suganthy et al.’s study in India which was performed on 100 subjects using MRI, the length of CC in males was significantly higher than females (72.6 ± 5.2 mm in male, 70.6 ± 4.0 mm in female). Suganthy also reported that only the length of CC increased with the increase of age [20].

In a study by Gupta et al. on the Indian population in 2009, their MRI showed that the length and width of CC was 7.57 cm and 3.27 cm, respectively in men and 7.1 cm and 2.59 cm, respectively in women and the splenial width values were 1.15 cm in men and 1.17 cm in women. Furthermore, Gupta reported that the length and widths of CC in the Indian population were greater than the Japanese, but lower than the Caucasian population [22].

Mourgela et al. [1] in Greece reported that inspite the positive linear correlation between the distance of the frontal and occipital poles of the brain to CC and brain dimensions, no significant linear correlation between the longitudinal dimensions of the brain and CC was observed.

In this study, there was no significant difference detected in CC diameters such as EZ, EZ/3. EZ/5, FG between genders. Our results were similar to the findings of Takeda’s study in Japan [21] and Tuncer’s study in Turkey [25].

Suganthy et al. (2003) and Mourgela et al. (2007) reported that only longitudinal dimension of CC is higher in males [1][20]. In our study, longitudinal dimensions of CC were higher than other studies such as Mourgela in Grecce [1], Takeda in Japan [21] and similar to Indians [20]. In addition, the width of CC was similar to other studies [1][21]. According to our findings, EZ, EZ/3, EZ/5 and FG were related to age groups. Takeda et al.‘s study has shown that the longitudinal diameter of CC increased with age, but the width of the rostrun and splenium and body decreased [21]. Suganthy et al. also reported that the length of CC increased with age but the other dimensions had no changes [20].

In this study, the CC dimensions increased in the 45- to 60-years age group. The finding is in contrast with other studies [1][20][21]. This difference may be due to the low number of 60-year-old subjects and age groups in our study.

A positive linear correlation was obvious between EZ and AB and between EZ and FG but not between EZ and CD. These findings are not similar to Mourgela’s study [1].

In our study, multivariant linear regression showed that AB had a strong positive correlation with EZ. Indeed, the longitudinal and vertical dimensions of the brain and the distance of the CC from the frontal and occipital poles had a positive linear correlation. However, there is a statistical relationship between the dimensions of the brain with the longitudinal dimensions of the CC. Thus, the various dimensions of the brain change correlatively with each other. So to preserve the symmetry of the human brain, the size and dimensions of CC can vary.

Regarding the correlation between the brain and CC dimensions in our study, we concluded that there is symmetry between the brain and the size of CC. This conclusion is similar to Estruch et al. [26] and Mourgela et al.’s study [1].

The differences on quantitative data of CC in various areas of the world which were seen in different studies may be due to racial/ethnic factors [21][22]. Therefore, further studies should be performed in order to estimate differences among various ethnicity/races and to establish the normal standard data in each population. There were limitations for our study; namely the low sample size and the low number of older than 60-year-old samples.
